# Simvastatin Inhibits CYR61 Expression in Orbital Fibroblasts in Graves' Ophthalmopathy through the Regulation of FoxO3a Signaling

**DOI:** 10.1155/2021/8888913

**Published:** 2021-01-21

**Authors:** Yi-Hsuan Wei, Shu-Lang Liao, Chia-Chun Wang, Sen-Hsu Wang, Wan-Chun Tang, Chang-Hao Yang

**Affiliations:** ^1^Department of Ophthalmology, National Taiwan University Hospital, Taipei, Taiwan; ^2^Graduate Institute of Clinical Medicine, College of Medicine, National Taiwan University, Taipei, Taiwan; ^3^Department of Ophthalmology, National Taiwan University College of Medicine, Taipei, Taiwan

## Abstract

Graves' ophthalmopathy (GO), which is characterized by orbital tissue inflammation, expansion, and fibrosis, is the ocular manifestation in 25% to 50% of patients with Graves' disease. As the pathology of GO is driven by autoimmune inflammation, many proinflammatory cytokines/chemokines, including TNF-*α*, IL-1*β*, IL-6, and CCL20, are crucial in the pathogenesis of GO to activate the orbital fibroblasts. Cysteine-rich protein 61 (CYR61), which is known to regulate cell proliferation, adhesion, and migration, plays a proinflammatory role in the pathogenesis of many inflammatory diseases, such as rheumatoid arthritis. CYR61 was considered a potential biomarker of GO in recent studies. Statins, which are cholesterol-lowering drugs, were found to reduce the risk of GO, probably through their anti-inflammatory and immunomodulatory effects. In this study, we established a link between CYR61 and statins in the pathogenesis and potential treatment for GO. Firstly, our data showed the overexpression of CYR61 in the orbital tissue (*n* = 4) and serum specimens (*n* = 6) obtained from the patients with inactive GO. CYR61 could induce the production of IL-6 and CCL20 in cultured GO orbital fibroblasts. The expression of CYR61 in cultured GO orbital fibroblasts was upregulated via TNF-*α* stimulation. Secondly, we pretreated cultured GO orbital fibroblasts using simvastatin, a statin, followed by TNF-*α* stimulation. The data revealed that simvastatin could inhibit TNF-*α*-induced CYR61 expression by modulating the activity of transcription factor FoxO3a. Our results provided insights into some cellular mechanisms that may explain the possible protective effects of simvastatin against the development of GO.

## 1. Introduction

Graves' disease (GD) is a common autoimmune disease in which stimulatory autoantibodies bind to the thyroid-stimulating hormone receptor (TSHR), resulting in increased thyroid gland activity and growth. Approximately 25 to 50% of patients with GD develop ocular manifestations, known as Graves' ophthalmopathy (GO), which is characterized by orbital inflammation, fibrosis, and tissue remodeling. The clinical signs of GO include conjunctival chemosis, periorbital soft tissue swelling, proptosis, restriction of eye movement, and a decrease in vision due to compressive optic neuropathy [1]. The pathogenesis of GO is not completely understood. TSHR and insulin-like growth factor receptor (IGF-1R) are the major autoantigens that are overexpressed in the orbital tissue of individuals with GO [2]. Orbital fibroblasts appear to play a crucial role in GO. They display a series of cell surface receptors and produce numerous proinflammatory cytokines and chemokines. These cause massive infiltration of T and B cells, hyaluronan accumulation, orbital adipogenesis, and tissue fibrosis [3].

Cysteine-rich protein 61 is also known as CCN1, which belongs to the CCN family of extracellular matrix-associated signaling protein. It has been shown to regulate angiogenesis, cell proliferation, adhesion, migration, and differentiation [4, 5]. Previous studies revealed that CYR61 plays a crucial role in the pathogenesis of proliferative diabetic retinopathy (PDR), as demonstrated by the increased expression of CYR61 in the vitreous humor of PDR patients [6]. The proinflammatory effect of CYR61 has also been revealed in the pathogenesis of rheumatoid arthritis (RA) [7]. In an expression study of orbital tissue, the CYR61 gene was found to be overexpressed in patients with GO [8]. Serum CYR61 levels were higher in GO patients than in controls. A genetic study suggested associations of single nucleotide polymorphisms (SNPs) in the CYR61 gene with GO [9]. Recently, serum CYR61 was proposed as an adjuvant biomarker of GO because patients with active GO showed significantly elevated CYR61 levels in the serum [10]. Therefore, it is assumed that CYR61 participates in the pathogenesis of GO.

Statins are a class of cholesterol-lowering drugs. They inhibit the enzyme hydroxymethylglutaryl-coenzyme A reductase, which plays an essential role in cholesterol synthesis. Recently, the pleiotropic effect of statins has been proposed as adjuvant anti-inflammatory therapy [11]. In recent literature, among patients with GD, the use of statins is associated with a reduced risk of developing GO [12]. It has been hypothesized that statins reduce GO risk mainly by modulating both apoptosis and autophagy activities [13]. Previous studies have proposed simvastatin, belonging to the class of drugs known as statins, as a potential therapeutic agent for RA because of its anti-inflammatory effect [14]. Simvastatin can inhibit CYR61 expression in rheumatoid arthritis synovial fibroblasts through the regulation of transcription factor Forkhead box O3 (FoxO3a) signaling [15].

In the present study, we first aimed to verify the overexpression of CYR61 in serum and orbital tissue from patients with GO. Secondly, in primary cultured GO orbital fibroblasts, we investigated the potential proinflammatory effect of CYR61 by accessing CYR61-induced IL-6 and CCL20, which are GO-associated proinflammatory cytokine and chemokine, respectively [16]. Thirdly, we assessed CYR61 expression in cultured cells after the stimulation of TNF-*α* which is one of the crucial cytokines in GO [17]. Furthermore, we aimed to evaluate the inhibitory effect of simvastatin on the expression of TNF-*α*-induced CYR61. Lastly, we tried to clarify the role of FoxO3a in the molecular mechanisms by which simvastatin inhibits CYR61 expression. We hypothesized that simvastatin has beneficial effects on GO through multiple mechanisms, including downregulation of CYR61 expression.

## 2. Materials and Methods

### 2.1. Orbital Tissue and Serum Collection

Orbital tissue specimens were collected from the surgical waste of six GO patients during orbital decompression. Healthy non-GO orbital tissue samples were obtained from six age- and sex-matched patients without inflammatory and thyroid disease who underwent cosmetic blepharoplasty. The patient characteristics are listed in Table 1. The GO patients were euthyroid with a clinical activity score of less than three for more than six months before the surgery. None of them had received radiotherapy or steroid pulse therapy in the past. Ten milliliters of venous peripheral blood was obtained from each GO and control subject and mixed with EDTA. The blood samples were centrifuged for 15 min to collect the serum for the measurement of the CYR61 level. Recruitment was performed at the Department of Ophthalmology of National Taiwan University Hospital. The protocol was approved by the Institutional Review Board of National Taiwan University Hospital (201709012RIND). Informed consent was obtained from all participants.

### 2.2. Orbital Fibroblast Culture and Stimulation

Orbital fibroblasts were cultivated as previously reported [18]. Orbital tissues were minced and placed in plastic culture dishes with Dulbecco's modified Eagle's medium (DMEM) supplemented with 20% fetal bovine serum (FBS) and 50 IU/ml penicillin-streptomycin (Gibco, Waltham, MA, USA), allowing orbital fibroblasts to grow out. Monolayers were covered with DMEM supplemented with 10% FBS and serially passaged with gentle trypsin/EDTA (Gibco, Waltham, MA, USA) treatment. They were incubated in a 37°C humidified incubator with a 5% CO_2_ environment.

The orbital fibroblasts were seeded into 6-well plates at a density of 2 × 10^5^ cells per well and stimulated at 90% confluence with recombinant human CYR61 (PeproTech, Rocky Hill, NJ, USA) and TNF-*α* (PeproTech, Rocky Hill, NJ, USA) or pretreated with simvastatin (TargetMol, Boston, MA, USA) followed by TNF-*α* stimulation. All experiments were performed with fibroblasts between the third and eighth passages from culture initiation. Three independent strains from different donors were used for the repeated experiments.

### 2.3. Immunohistochemical and Immunofluorescence Staining

The specimens collected from the GO patients and healthy controls were fixed in 4% buffered formalin and embedded in paraffin. Tissue cross-sections were prepared at a 5 *μ*m thickness for immunohistochemical staining. After blocking the endogenous peroxidase activity in 3% H_2_O_2_, sections were incubated overnight with anti-CYR61 antibodies (ab24448, Abcam, Cambridge, MA, USA). The sections were washed with phosphate-buffered saline (PBS) and incubated with biotinylated secondary antibodies for 20 min. After washing with PBS, the sections were incubated with horseradish peroxidase-conjugated streptavidin for 20 min and processed with a DAB Substrate Kit (SK-4100, Vector Laboratories, Burlingame, CA, USA). Finally, they were counterstained with hematoxylin.

The orbital fibroblasts were cultured on six-well plates containing glass coverslips, and the cells were fixed and blocked for the immunofluorescence assay. The coverslips were incubated with FoxO3a primary antibodies (#2497, Cell Signaling Technology, Beverly, MA, USA) overnight at 4°C. After three washes, the coverslips were processed with secondary antibodies conjugated to Alexa Fluor 488 (A11034, Invitrogen, Carlsbad, CA, USA). The nuclei were counterstained using DAPI (H-1500, Vector Laboratories, Burlingame, CA, USA). The glass slides were observed using a confocal microscope (LSM 800, Carl Zeiss), and representative cells were imaged and analyzed.

### 2.4. Real-Time PCR

Total RNA was extracted from the orbital tissue or cultured orbital fibroblasts using TRIzol (Invitrogen, Carlsbad, CA, USA). Complementary DNA was synthesized using 1 *μ*g of RNA according to the manufacturer's instructions for the iScript cDNA Synthesis Kit (Bio-Rad, Hercules, CA, USA). Real-time PCR was performed on a thermocycler (StepOne Real-Time PCR System; Applied Biosystems, Foster City, CA, USA) using SYBR Green Master Mix (Applied Biosystems, Foster City, CA, USA). All PCRs were performed in triplicate. The primers used in this study were HP101467 for CYR61, HP100427 for IL-6, and HP100003 for GAPDH (Sino Biological, Wayne, PA, USA); and the primers for CCL20 were 5′-GTG CGC AAA TCC AAA ACA GA-3′ (forward) and 5′-CCA ACC CCA GCA AGG TTC TT-3′ (reverse). The mRNA levels of each target gene were normalized to GAPDH and represented as fold changes.

### 2.5. Enzyme-Linked Immunosorbent Assay (ELISA)

The concentration of serum CYR61 was measured with ELISA kits (R&D Systems, Minneapolis, MN, USA) according to the manufacturer's instructions. ELISA kits for IL-6 (BioLegend, San Diego, CA, USA) and CCL20 (R&D Systems, Minneapolis, MN, USA) were used to detect the protein levels in the culture supernatant of orbital fibroblasts according to the manufacturer's protocols. Absorbance was read at 450 nm, and sample values were compared with the standard curve. The mean value of the triplicate samples was reported.

### 2.6. Western Blot Analysis

Proteins were extracted from tissue homogenates and cell lysates. For the western blot of subcellular fractionation, nuclear and cytoplasmic fractions were prepared using an NE-PER nuclear and cytoplasmic extraction kit (Thermo Fisher Scientific, Rockford, IL, USA) according to the manufacturer's instructions. After collecting the protein samples, equal amounts of protein (50 *μ*g) were boiled in the sample buffer. The protein samples were separated in a 10% sodium dodecyl sulfate-polyacrylamide gel and transferred to a nitrocellulose membrane. The blots were blocked with 4% bovine serum albumin for 1 h at room temperature and then probed with primary antibodies at 4°C overnight. Antibodies against CYR61, FoxO3a, and phosphorylated FoxO3a were purchased from Cell Signaling Technology (Beverly, MA, USA). Immunoreactive bands were detected with horseradish peroxidase-conjugated secondary antibodies and developed using enhanced chemiluminescence detection (MilliporeSigma, Burlington, MA, USA) and exposure to X-ray film. The relative value of each immunoreactive band was quantified using ImageJ software and normalized to the levels of reference molecules.

### 2.7. Cell Transfection Assay

The role of FoxO3a in TNF-*α*-induced CYR61 expression was studied by transfecting orbital fibroblasts with FoxO3a cDNA. The plasmid with FoxO3a cDNA construct (pcDNA-flag-FoxO3a) was purchased from Addgene (Cambridge, MA, USA).

For transient transfections, the orbital fibroblasts were transfected for 24 h using Lipofectamine 2000 (Invitrogen, Carlsbad, CA, USA) according to the manufacturer's instructions and then seeded for the experiment. The infection efficiency was validated using western blot assays.

### 2.8. Chromatin Immunoprecipitation (ChIP) Assay

ChIP assay was performed using the EZ-Magna ChIP HiSens kit (Cat. No. 17-10461, Sigma-Aldrich, St. Louis, MO, USA). The protein-DNA complexes from cultured orbital fibroblasts were precipitated using an anti-FoxO3a antibody (NBP2-16521, Novusbio, Littleton, CO, USA). The cross-linked protein-DNA complexes were reversed, and the DNA was purified for real-time PCR according to the manufacturer's instructions. The primers specific for the region of the CYR61 promoter flanking the FoxO3a binding sites were as follows: forward 5′-CCAACCAGCATTCCTGAGAT-3′ and reverse 5′-CGTATAAAAGGCGGGCTCC-3′ according to the reference [15].

### 2.9. Statistical Analyses

At least three cell strains from different individuals were used in all experiments, and sample assays were done in triplicate. The experimental results are shown as the mean ± SD calculated from normalized measurements. Analysis of variance or Student's *t*-test was used to determine statistical significance (*p* < 0.05) using GraphPad Prism 8.4.2 (GraphPad Software, San Diego, CA, USA).

## 3. Results

### 3.1. CYR61 Expression Increased in the Serum and Orbital Tissue from GO Patients

**Figure 1:**
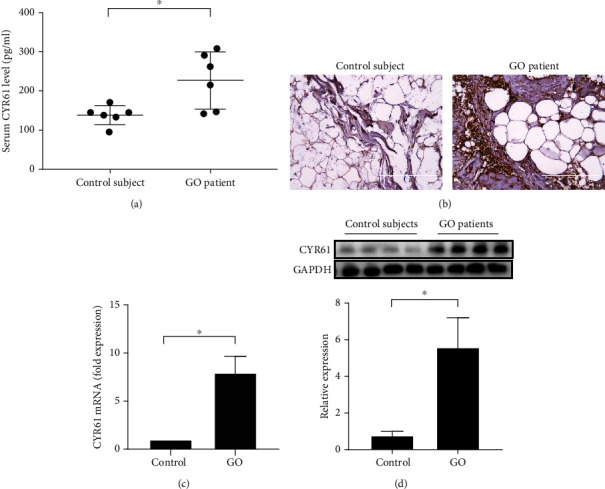
The expressions of CYR61 in the serum and orbital tissue from healthy controls and GO patients. (a) Serum CYR61 concentrations in control subjects and GO patients. (b) Representative images of immunohistochemical staining using an anti-CYR61 antibody (brown) in orbital connective tissue from a control subject and GO patient. (c) The expression of CYR61 mRNA in orbital connective tissue from control subjects and GO patients was detected by real-time PCR (*n* = 4 in each group). (d) The expression of CYR61 protein in orbital connective tissue from control subjects and GO patients was detected by western blot analysis (*n* = 4 in each group). Data are presented as mean ± SD. ^∗^*p* < 0.05.

To identify the expression of CYR61 in the peripheral blood, serum from control subjects and GO patients was examined via ELISA. The serum concentration of CYR61 was higher in the GO patients than the control subjects (Figure 1(a)). We next examined the expression of CYR61 in the orbital connective tissue. Immunohistochemistry staining revealed that CYR61 expression was higher in the orbital connective tissue from GO patients than in that from control subjects (Figure 1(b)). We further quantified the difference in CYR61 expression in orbital tissue between GO patients and control subjects using real-time PCR and western blot analysis. Four subjects were randomly chosen from each group. The expression levels of CYR61 mRNA and protein in orbital tissue were significantly higher in patients with GO than in controls (Figures 1(c) and 1(d)). These data validated that CYR61 could be a biomarker for GO, which is worthy of further investigation.

### 3.2. CYR61 Induced the Production of IL-6 and CCL20 in GO Orbital Fibroblasts

**Figure 2:**
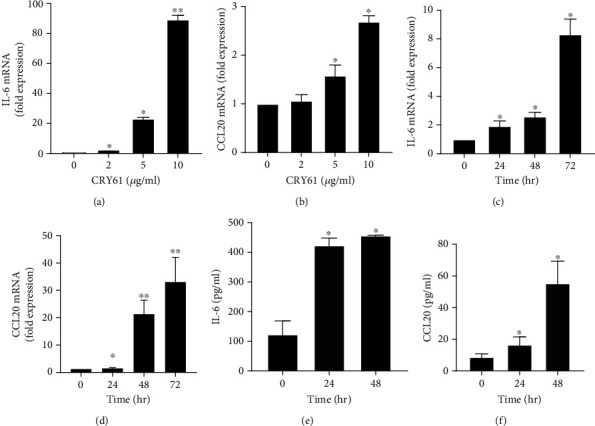
Dose- and time-dependent effects of CYR61 on the production of IL-6 and CCL20 in GO orbital fibroblasts. (a, b) GO orbital fibroblasts were treated with different doses (0, 2, 5, and 10 *μ*g/ml) of CYR61 for 24 h. IL-6 mRNA (a) and CCL20 mRNA (b) expressions were examined by real-time PCR. (c) GO orbital fibroblasts were treated with 2 *μ*g/ml CYR61 for 0, 24, 48, or 72 h. IL-6 mRNA expression was determined by real-time PCR. (d) GO orbital fibroblasts were treated with 5 *μ*g/ml CYR61 for 0, 24, 48, or 72 h. CCL20 mRNA expression was determined by real-time PCR. (e, f) GO orbital fibroblasts were treated with 5 *μ*g/ml CYR61 for 24 and 48 h. The protein levels of IL-6 (e) and CCL20 (f) in the culture supernatant were measured by ELISA. Data are presented as mean ± SD from at least three independent experiments. ^∗^*p* < 0.05, ^∗∗^*p* < 0.01.

To evaluate the proinflammatory effect of CYR61 on orbital fibroblasts, primary fibroblasts derived from the orbital tissue of GO patients were treated with different concentrations of CYR61 for different time points. Many proinflammatory molecules play important roles in the pathogenesis of GO, including the proinflammatory cytokine, IL-6, and proinflammatory chemokine, C-C chemokine ligand 20 (CCL20) [16]. We tested the potential effect of CYR61 on the expression of these two molecules in the orbital fibroblasts of GO patients. The results showed that CYR61 significantly induced IL-6 and CCL20 mRNA expression in a dose- and time-dependent manner (Figures 2(a)–2(d)). Consistent with these results, the ELISA revealed that concentrations of IL-6 and CCL20 in the orbital fibroblast culture supernatant were significantly increased after CYR61 stimulation (Figures 2(e) and 2(f)). This indicates that CYR61 could promote proinflammatory cytokine/chemokine production and trigger a future inflammatory process in the pathogenesis of GO.

### 3.3. TNF-*α*-Stimulated CYR61 Production in GO Orbital Fibroblasts

**Figure 3:**
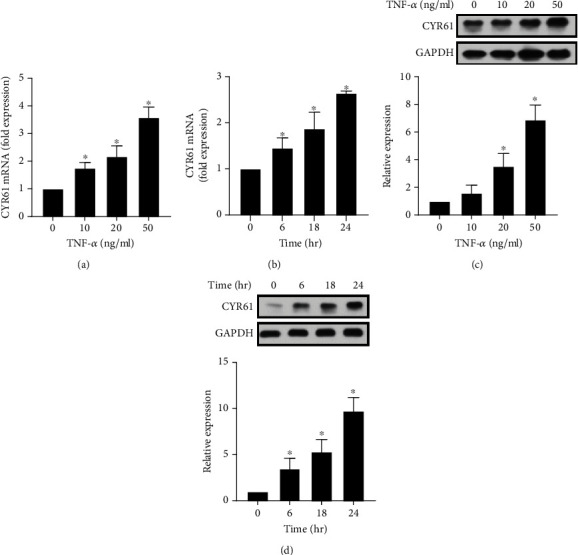
Dose- and time-dependent effects of TNF-*α* on the production of CYR61 in GO orbital fibroblasts. (a) GO orbital fibroblasts were treated with different concentrations of TNF-*α* (0, 10, 20, and 50 ng/ml) for 6 h. CYR61 mRNA expression was detected by real-time PCR. (b) GO orbital fibroblasts were treated with 10 ng/ml TNF-*α* for 0, 6, 18, and 24 h. CYR61 mRNA expression was detected by real-time PCR. (c) GO orbital fibroblasts were treated with different concentrations of TNF-*α* (0, 10, 20, and 50 ng/ml) for 18 h. CYR61 protein production was examined by western blot analysis. (d) GO orbital fibroblasts were treated with 20 ng/ml TNF-*α* for 0, 6, 18, and 24 h. CYR61 protein production was examined by western blot analysis. Data are presented as mean ± SD of at least three independent experiments. ^∗^*p* < 0.05.

To investigate the possible mechanism of CYR61 overexpression in the orbital tissue from patients with GO, we measured the synthesis of CYR61 in GO orbital fibroblasts stimulated by TNF-*α*. The results showed that CYR61 mRNA expression was significantly increased after TNF-*α* stimulation in a dose- and time-dependent manner (Figures 3(a) and 3(b)). We further examined the protein expression of CYR61 in GO orbital fibroblasts treated with different concentrations of TNF-*α*. The results revealed that the synthesis of CYR61 significantly increased upon 20 ng/ml TNF-*α* stimulation (Figure 3(c)). The protein levels of CYR61 also increased in a time-dependent manner (Figure 3(d)). Since TNF-*α* is a major inflammatory cytokine in the orbital tissue from patients with GO [19], our in vitro data indicated that the overexpression of CYR61 could be stimulated by TNF-*α* in GO orbital tissue.

### 3.4. Simvastatin Inhibited TNF-*α*-Induced CYR61 Production in GO Orbital Fibroblasts

**Figure 4:**
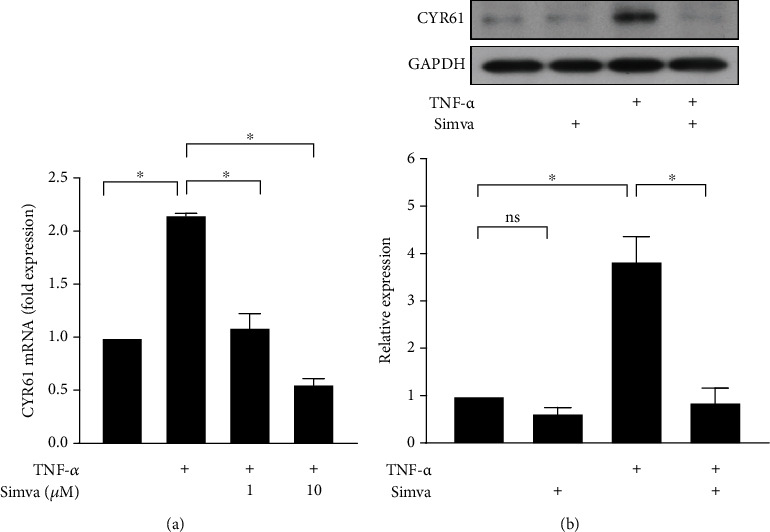
Effects of simvastatin on TNF-*α*-induced CYR61 production in GO orbital fibroblasts. (a) GO orbital fibroblasts were stimulated with 20 ng/ml TNF-*α* for 24 h with or without a 3 h pretreatment with simvastatin (1 *μ*M or 10 *μ*M). CYR61 mRNA expression was examined by real-time PCR. (b) GO orbital fibroblasts were stimulated with 20 ng/ml TNF-*α* for 24 h with or without a 3 h pretreatment with simvastatin (10 *μ*M). CYR61 protein production was determined by western blot analysis. Data are presented as mean ± SD of at least three independent experiments. ^∗^*p* < 0.05.

To determine whether simvastatin could inhibit CYR61 expression in GO orbital fibroblasts, the cells were stimulated with 20 ng/ml TNF-*α* for 24 h with or without a 3 h pretreatment of simvastatin (1 *μ*M or 10 *μ*M). The results of the real-time PCR revealed that TNF-*α*-induced CYR61 mRNA expression was significantly inhibited by simvastatin (Figure 4(a)). The results of western blot analysis showed that the protein levels of TNF-*α*-induced CYR61 in GO orbital fibroblasts were also significantly inhibited by simvastatin (Figure 4(b)). These results indicated that inhibition of CYR61 expression could be one of the mechanisms for simvastatin to prevent the inflammatory process in GO.

### 3.5. Simvastatin Inhibited TNF-*α*-Induced CYR61 Production through the Regulation of FoxO3a Signaling

**Figure 5:**
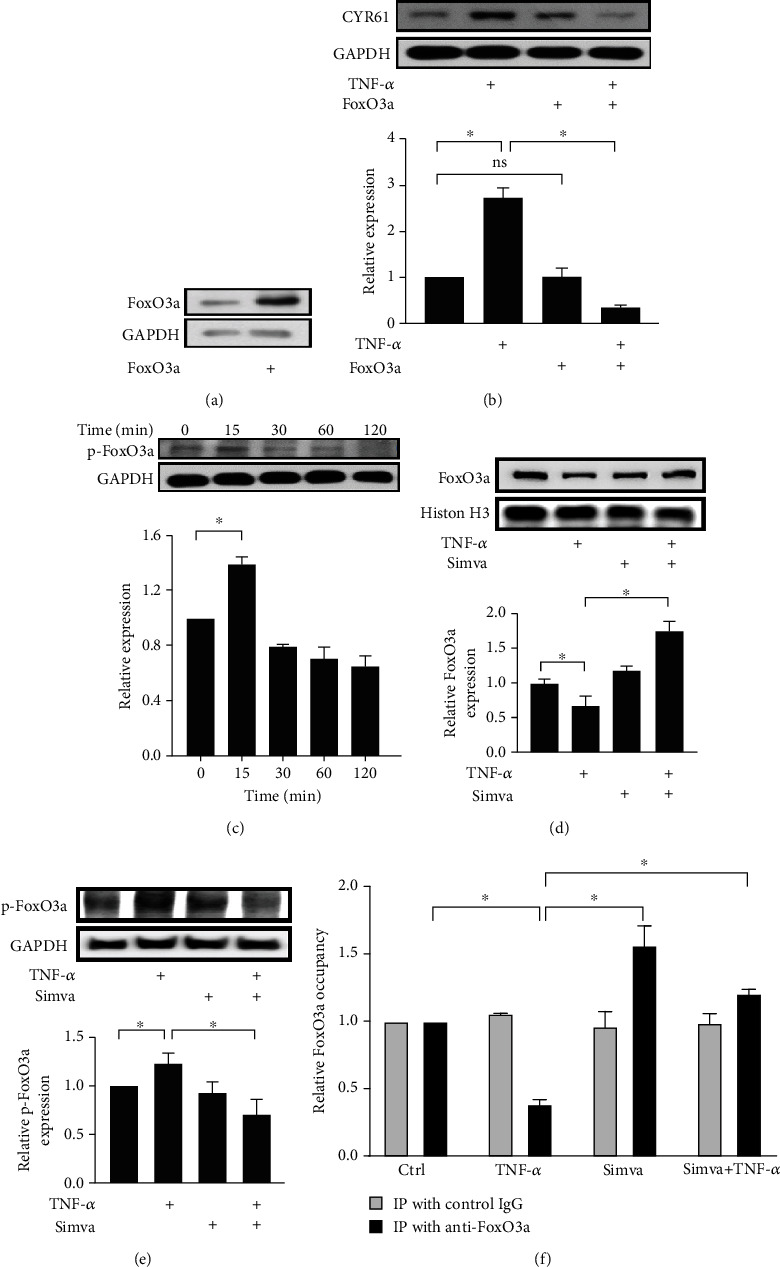
Roles of FoxO3a in TNF-*α*-induced CYR61 expression and effects of simvastatin on FoxO3a. (a) After transfecting GO orbital fibroblasts with FoxO3a-expressing plasmid, overexpression of FoxO3a in transfected orbital fibroblasts was confirmed by western blot analysis. (b) Original orbital fibroblasts and transfected orbital fibroblasts with overexpression of FoxO3a were exposed to 20 ng/ml TNF-*α* for 24 h with or without pretreatment with 10 *μ*M simvastatin for 3 h. CYR61 protein production was assessed by western blot analysis. (c) GO orbital fibroblasts were incubated with 20 ng/ml TNF-*α* for the indicated time points. The p-FoxO3a protein in total cell lysates was examined by western blot analysis. (d, e) GO orbital fibroblasts were exposed to 20 ng/ml TNF-*α* for 15 min with or without pretreatment with 10 *μ*M simvastatin for 1 h. The levels of FoxO3a in the nuclear fractions (d) and p-FoxO3a in the cytoplasmic fractions (e) were measured by western blot analysis. (f) GO orbital fibroblasts treated with 20 ng/ml TNF-*α* for 15 min with or without pretreatment with 10 *μ*M simvastatin were subjected to chromatin immunoprecipitation (IP) with anti-FoxO3a antibodies. The immunoprecipitated DNA were evaluated by real-time PCR using primers for the region of the CYR61 promoter flanking the FoxO3a binding site. Data are presented as mean ± SD of at least three independent experiments. ^∗^*p* < 0.05.

Previous studies have shown the role of FoxO3a in CYR61 expression and its modulation by simvastatin in cultured osteoblasts [20] or synovial fibroblasts [15]. We explored whether simvastatin inhibited CYR61 expression through the regulation of FoxO3a signaling in GO orbital fibroblasts. Firstly, to investigate how FoxO3a regulates CYR61 expression in GO orbital fibroblasts, we successfully transfected the cells with FoxO3a-expressing plasmid via cell transfection assay (Figure 5(a)). The level of endogenous CYR61 was not changed in the orbital fibroblasts transfected with FoxO3a, but TNF-*α*-induced CYR61 production was suppressed in these cells (Figure 5(b)). The data implied that FoxO3a is a negative regulator of CYR61 expression in orbital fibroblasts.

Secondly, the results of western blot analysis showed enhanced expression of phosphorylated FoxO3a (p-FoxO3a) in GO orbital fibroblasts after TNF-*α* treatment. The peak level of p-FoxO3a appeared at 15 min after the treatment (Figure 5(c)). Phosphorylation of FoxO3a results in cytoplasmic translocation of FoxO3a and reduced biological activity of FoxO3a [21]. Therefore, we further investigated whether simvastatin could inhibit TNF-*α*-induced phosphorylation of FoxO3a in GO orbital fibroblasts. Cultured GO orbital fibroblasts were stimulated with 20 ng/ml TNF-*α* for 15 min with or without pretreatment with 10 *μ*M simvastatin. The levels of FoxO3a in the nuclear fractions and p-FoxO3a in the cytoplasmic fractions were measured via western blot analysis. The results demonstrated that simvastatin maintained the nuclear localization of FoxO3a after TNF-*α* treatment (Figure 5(d)). At the same time, simvastatin inhibited TNF-*α*-induced p-FoxO3a expression in the cytoplasm (Figure 5(e)).

**Figure 6:**
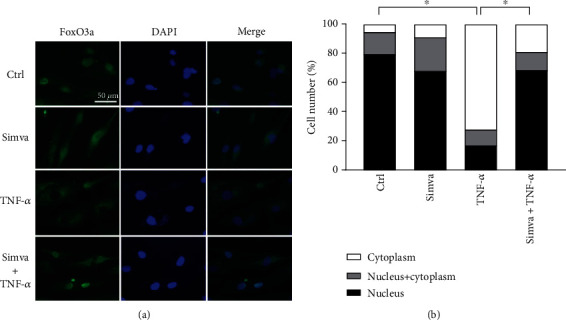
Simvastatin inhibits TNF-*α*-induced FoxO3a translocation from the nucleus to the cytoplasm. (a) Immunofluorescence staining of cultured GO orbital fibroblasts stimulated with 20 ng/ml TNF-*α* for 15 min with or without a 1 h pretreatment with 10 *μ*M simvastatin. It revealed increased FoxO3a expression (green) in the cytoplasm upon TNF-*α* stimulation. The cytoplasmic translocation of FoxO3a induced by TNF-*α* was inhibited by simvastatin. (b) The percentage of cells in which FoxO3a was exclusively located in the nucleus (black bars), exclusively in the cytoplasm (white bars), or diffuse in the nucleus and the cytoplasm (gray bars) is shown. The statistical analysis of FoxO3a in the nucleus and in the cytoplasm was performed. ^∗^*p* < 0.05.

Moreover, the ChIP assay was performed to investigate how FoxO3a signaling specifically controls CYR61 expression. GO orbital fibroblasts stimulated with TNF-*α* with or without simvastatin pretreatment were subjected to chromatin immunoprecipitation (IP) with anti-FoxO3a antibodies. The immunoprecipitated DNA were evaluated by real-time PCR using primers for the region of the CYR61 promoter flanking the FoxO3a binding site. The results of the ChIP assay revealed that TNF-*α* stimulation reduced FoxO3a binding to the CYR61 promoter while simvastatin may restore the event (Figure 5(f)). Immunofluorescence staining of cultured orbital fibroblasts also showed the cytoplasmic translocation of FoxO3a induced by TNF-*α*, which could be inhibited by simvastatin (Figure 6).

## 4. Discussion

The manifestation of GO is a chronic inflammatory process of the orbital tissues that can result in disfigurement and potential visual impairment. Inflammatory cytokines play an important role in the pathogenesis of GO. TNF-*α* is a potent proinflammatory cytokine that is produced by T cells, natural killer cells, mast cells, and fibroblasts. It was found to be overexpressed in the orbital connective tissue [19, 22] and serum [23, 24] from the patients with GO. Cultured fibrocytes have been shown to produce TNF-*α* in response to TSH [25]. In our study, we stimulated GO orbital fibroblasts with TNF-*α* to induce the synthesis of CYR61, which may further induce other cytokines, as shown in many studies [26]. We demonstrated that CYR61 induced the expression of IL-6 and CCL20, which are both biomarkers for GO [16, 27]. Fang et al.'s study reported that CCL20 produced by activated fibrocytes may recruit Th17 cells to promote inflammation and play a role in the pathogenesis of GO [28]. The actions of the proinflammatory cytokines/chemokines, such as IL-6 and CCL20, can subsequently lead to inflammation and tissue remodeling such as that occurring in GO [29]. Further in vitro and in vivo investigations with a larger sample size are necessary to understand the role of CYR61 in the complex interactions between various cytokines and inflammatory cells in the pathogenesis of GO.

CYR61, a product of an immediate early gene, is known to regulate cell proliferation, adhesion, and migration [5]. Recent studies have indicated that CYR61 also acts as a proinflammatory factor involved in the pathogenesis of many inflammatory diseases [26]. In rheumatoid arthritis, CYR61 was found to be overexpressed in the synovial tissue of patients and was able to induce the expression of IL-6, CCL20, IL-1*β*, and MMP-3 in cultured fibroblast-like synoviocytes from patients [15, 30–32]. In diabetic retinopathy, CYR61 promotes inflammation by inducing the expression of MCP-1 in chorioretinal vascular endothelial cells [33]. In recent years, studies have presented the association between CYR61 and GO. Firstly, CYR61 expression was increased in the orbital tissue of patients with GO compared with healthy individuals [8]. Secondly, four SNPs in the CYR61 gene were found to be associated with GO [9]. Thirdly, CYR61 expression is induced in human dermal fibroblasts after exposure to cigarette smoke, which is a strong risk factor for GO [34]. Moreover, serum CYR61 showed good potential as a biomarker for active GO [10]. Our study validated the overexpression of CYR61 in the orbital tissue and serum in GO patients. We further demonstrated that TNF-*α* induced CYR61 synthesis, which is a possible mechanism of CYR61 overexpression in GO.

Statins are commonly used to prevent coronary artery disease and stroke by reducing low-density lipoprotein cholesterol levels. Recently, the pleiotropic anti-inflammatory effects of statins have been proposed [11, 35]. Various studies suggested that statins could be used as a new adjuvant anti-inflammatory therapy [35–40]. Furthermore, the possible protective role of statins in GO has been proposed recently [41]. Statin usage is believed to be associated with a reduced risk of developing GO among patients with GD in a large cohort study [12]. They found that patients who used statins for at least 60 days during the period of observation had a 40% lower risk of developing GO. In contrast, no significant association was found for the use of other nonstatin cholesterol-lowering drugs [12]. The precise molecular mechanisms by which statins reduce GO risk are not fully established. Some evidence suggests that statins may modulate both apoptosis and autophagy activities in patients with GD [13]. This elucidation was based on the involvement of cellular apoptosis and autophagy in the pathogenesis of GO [42, 43] and the ability of statins to modulate apoptosis and autophagy in human atrial fibroblasts, airway fibroblasts, and murine osteoblasts [44–46]. In addition, a recent study revealed that simvastatin may inhibit adipogenesis, as well as the expression of early and late adipogenic genes in 3T3-L1 preadipocytes and human orbital fibroblasts [47]. The authors found that cigarette smoke extract alone or in combination with a differentiation cocktail may induce the expression of early adipogenic gene CYR61, late adipogenic genes SCD1 and PPAR-*γ*, and cytokines IL-6 and IL-1*β*. Simvastatin successfully suppressed all of these genes and adipocyte differentiation [47].

In addition to the abovementioned mechanisms, our study suggested that simvastatin may prevent GO development by inhibiting the expression of TNF-*α*-induced CYR61. Since CYR61 is a multifunctional gene with roles in adipogenesis, inflammation, and fibrosis [26, 48, 49] that may all contribute to the pathogenesis of GO, it could be a therapeutic target in GO. Anti-inflammatory effects similar to those of statins have been presented in other cell types such as osteoblasts and synovial fibroblasts in patients with rheumatoid arthritis [14, 15]. The cytokine-induced CYR61 expressions in these cells were suppressed in the presence of simvastatin. This finding indicates that simvastatin may have potential as a therapeutic agent for rheumatoid arthritis by targeting CYR61 [50]. In the same way, our study provided insight into the possible mechanism for simvastatin as a potential adjunctive treatment for GO.

Among the multiple binding sites of the CYR61 gene promoter, there is a site for the transcription factor FoxO3a, which functions as a transcriptional repressor of CYR61 [51]. FoxO3a, which belongs to the FoxO family of transcription factors, plays important roles in various cellular processes including proliferation, metabolism, apoptosis, and stress tolerance [52]. The activity of FoxO3a proteins can be regulated by posttranslational phosphorylation modification [53]. Phosphorylation modification regulates FoxO3a activity via a cytoplasmic-nuclear shuttling mechanism. Akt is the major kinase to phosphorylate FoxO3a at threonine (Thr)32, serine (Ser)253, and Ser315. Akt-dependent phosphorylation promotes the association of FoxO3a with 14-3-3 proteins in the nucleus, which in turn inhibits FoxO3a and DNA binding, leading to the exclusion of FoxO3a from the nucleus and reduced FoxO3a activity [53, 54]. In our study, we found that FoxO3a might be a negative regulator of CYR61 expression because the forced expression of FoxO3a inhibited TNF-*α*-induced CYR61 synthesis in orbital fibroblasts. TNF-*α* induced phosphorylation of FoxO3a and cytoplasmic translocation of p-FoxO3a, which subsequently diminished the activity of FoxO3a. On the contrary, simvastatin could retain FoxO3a in the nucleus, possibly through the inhibition of phosphorylation. Our results demonstrated that simvastatin inhibits CYR61 by modulating FoxO3a activity and proposed another possible mechanism to explain the beneficial effect of simvastatin on GO.

This study has some limitations. Firstly, the GO orbital tissues and serum were obtained from patients with inactive GO because of the surgical indication. Further studies should be conducted using specimens from patients with active GO. Secondly, we had a small number of patients and control subjects from whom serum and orbital tissues were collected. We may need a larger sample size to validate our results in the future. Thirdly, most of the experiments were conducted with orbital fibroblasts only from inactive GO patients. Further investigations are needed to compare the results among active GO, inactive GO, and control orbital fibroblasts.

In conclusion, this study provided insights into some cellular mechanisms that may explain the possible protective effects of simvastatin against the development of GO. The mechanisms of statins for the treatment of GO could be complex, and one of them may be associated with the inhibition of CYR61 actions. We believe that the effects of statins on GO are encouraging and deserve more investigation. Further prospective studies are needed to establish the clinical implications of statins in the prevention or management of GO.

## Figures and Tables

**Table 1:** Characteristics of patients with Graves' ophthalmopathy (GO) and non-GO control subjects included in this study.

	GO patients (*n* = 6)	Control subjects (*n* = 6)
Mean age (years)	44.0 ± 12.3	42.7 ± 11.3
Sex (male/female)	2/4	2/4
Smoking (*n*)	2	1
Duration of GD (months)	46.3 ± 9.7	—
Duration of GO (months)	33.8 ± 8.0	—
Previous orbital irradiation	0	—
Previous steroid pulse therapy	0	—
Euthyroid	6	6
TSH receptor antibodies	6	0
Clinical activity score	1.5 ± 0.5	

GD = Graves' disease; TSH = thyroid-stimulating hormone.

## Data Availability

The data are available from the corresponding author on reasonable request.
